# Risk of diabetes mellitus in HIV-infected patients receiving highly active antiretroviral therapy: A nationwide population-based study

**DOI:** 10.1097/MD.0000000000012268

**Published:** 2018-09-07

**Authors:** Shih Ping Lin, Chun-Ying Wu, Chang-Bi Wang, Tsai-Chung Li, Nai-Ying Ko, Zhi-Yuan Shi

**Affiliations:** a Department of Internal Medicine, Taichung Veterans General Hospital, Taichung; b National Cancer Institute, National Health Research Institute, Miaoli; c Graduate Institute of Clinical Medicine, National Yang-Ming University, Taipei; d Department of Life Sciences, National Chung Hsing University; e Department of Public Health, College of Public Health, China Medical University; f Department of Nursing, Central Taiwan University of Science and Technology; g Graduate Institute of Public Health, College of Public Health, China Medical University, Taichung; h Department of Nursing, College of Medicine, National Cheng Kung University and Hospital, Tainan, Taiwan.

**Keywords:** cohort study, comorbidity, hyperglycemia, metabolic syndrome, propensity score matching

## Abstract

Previous studies have shown that the incidence of diabetes mellitus (DM) has increased in human immunodeficiency virus (HIV)-infected patients with long-term exposure to highly active antiretroviral therapy (HAART). However, the factors associated with DM among HIV-infected patients in Asia remain unclear in the HAART era.

A nationwide cohort study

Data from Taiwan's National Health Insurance Research Database (NHIRD) between 2000 and 2010 were used to investigate the incidence of and factors associated with DM among HIV-infected patients. Propensity score matching was conducted to match 4797 patients receiving HAART (HAART cohort) with 4797 patients not receiving HAART (non-HAART cohort). HAART use was treated as a time-dependent variable in a Cox regression model.

HAART cohort had a significantly higher 10-year incidence of DM (7.16%; 95% confidence interval [CI], 4.30%–10.03%) than non-HAART cohort (2.24%; 95% CI, 1.28%–3.20%) (*P* < .001). After adjusting for age, gender, and comorbidities, receiving HAART was associated with an increased incidence of DM, with a subdistribution hazard ratio (sHR) of 2.39 (95% CI, 1.65–3.45). Hypertension (sHR = 5.27; 95% CI, 3.21–8.65), gout (sHR = 2.39; 95% CI, 1.38–4.16), and hepatitis C virus (HCV) infection (sHR = 2.43; 95% CI, 1.28–4.61) were significantly associated with a higher risk of DM. Sensitivity analyses showed exposure to HAART remained significantly associated with an increased risk of DM, particularly in those without pre-existing hypertension, gout, or HCV infection.

Exposure to HAART increased the risk of DM in HIV-infected Taiwanese patients, particularly in those without pre-existing hypertension, gout, or HCV infection.

## 1 Introduction

With the advent of highly active antiretroviral therapy (HAART), human immunodeficiency virus (HIV) infection has become a manageable chronic medical condition;^[1]^ however, the impact of metabolic complications related to long-term exposure to HAART and aging on the long-term successful management of HIV infection remains to be monitored and investigated.^[2–6]^ It is observed that the risk of diabetes mellitus (DM) among HIV-infected patients has increased with long-term exposure to HAART.^[7]^ DM will subsequently increase the risks of coronary artery disease, stroke, peripheral vascular disease, retinopathy, chronic kidney disease, and dementia. Previous studies hypothesized that interactions between HIV infection, HAART, and inflammation could significantly contribute to the risk of DM;^[9,10]^ moreover, older age, male gender, and hepatitis C virus (HCV) co-infection were also identified to be related to development of DM.

The annual incidence of DM among HIV-infected patients may range from 0.42% to 4.7%, depending on the study populations, the type of HAART and its exposure duration, and definition of DM used.^[11–15]^ Although previous cohort studies reported a higher incidence of DM among HIV-infected patients receiving HAART, most of these cohort studies were conducted in predominately Caucasian populations; population-based studies of DM among HIV-infected patients are rarely conducted in non-Caucasian HIV-infected patients. The interactions between traditional risk factors and HAART on the risk of DM among HIV-infected patients remain unclear. In this nationwide population-based cohort study, we aimed to investigate the association of HAART with the subsequent development of type 2 DM among HIV-infected Taiwanese patients.

## 2 Methods

### 2.1 Database

Data for the nationwide 2-group cohort study were derived from all HIV-infected patients in Taiwan's National Health Insurance Research Database (NHIRD). A nationwide surveillance system for HIV infection was established by the Department of Health (now the Ministry of Health and Welfare) in Taiwan in 1989. In Taiwan, HAART has been made available to all HIV-infected patients through the National Health Insurance (NHI) program since 1997, which makes the data on all HIV-infected patients in the NHIRD credible. During the study period (January 1, 2000 to December 31, 2010), the most common antiretroviral regimens used in Taiwan included 2 nucleoside analog reverse-transcriptase inhibitors (NRTIs) plus non-nucleoside reverse-transcriptase inhibitors (nNRTIs) or boosted or unboosted protease inhibitors (PIs). Integrase strand transfer inhibitors were not available in clinical use in Taiwan until July 2011.

Patients with newly diagnosed HIV infection who were antiretroviral-naive (International Classification of Disease, Ninth Revision [ICD-9] codes 042) were identified from the NHIRD between January 1, 2000 and December 31, 2010. Patients were classified into HAART cohort, which consisted of patients who received HAART for more than 170 days within 6 months of the diagnosis of HIV infection. In total, 18,451 patients were identified with newly diagnosed HIV infection from 2000 to 2011; patients who received the diagnosis of HIV infection before 2001 and after 2009 were excluded; and 7264 HIV-infected patients (39.4% of total), who received HAART therapy for more than 170 days within 6 months of the initial diagnosis of HIV infection, were eligible for inclusion. Type 1 DM was excluded from this study. With further exclusion of the patients with a diagnosis of DM made before and within 1 year after HIV diagnosis, the remaining 4797 patients were included in the HAART cohort. Patients with DM developing within the 1st year of the follow-up period were not included due to the difficulties in establishing the relationship between HIV infection and HAART and the subsequent development of DM.

**Figure 1:**
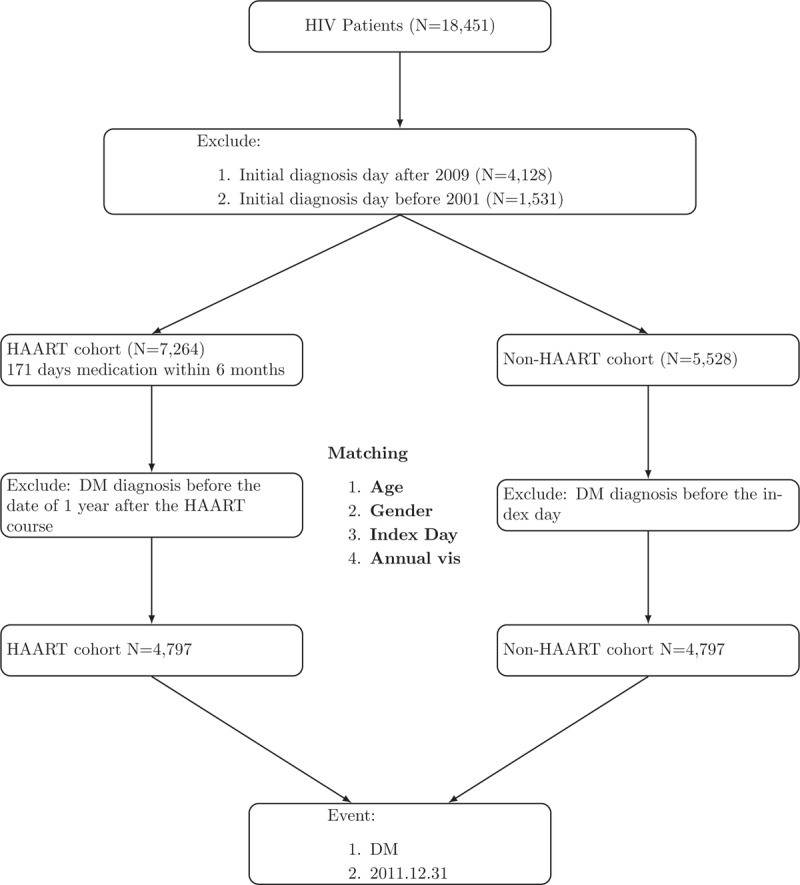
Flowchart of patient enrollment.

Among those who were newly diagnosed with HIV infection, 5528 patients (30.0%) who did not receive any HAART during the entire follow-up period were included in the non-HAART cohort; of them 4797 patients were identified as controls after matching for age, gender, numbers of annual visits, and index date of HIV diagnosis (Fig. 1). The index date of HIV diagnosis for the 2 cohorts was assigned as the date of 1 year after the initial HIV diagnosis.

Prior comorbidities were classified as those existing prior to the index date of HIV diagnosis, including hypertension, gout, chronic hepatitis B virus (HBV) infection, HCV infection, and cancer. The end of the follow-up period for the analyses was the date of DM diagnosis, termination of enrollment in the NHI, or death, or December 31, 2011, whichever occurred first. Follow-up data were available for a minimum of 1 year for all subjects. The Ethics Committee of Taichung Veterans General Hospital approved this study as the analysis in the study only included de-identified secondary data for research purposes.

### 2.2 Definition of outcome

The primary outcome was DM ([ICD-9] codes 250.X). The diagnosis of DM made during the follow-up period required meeting one of the following criteria: 1 or more inpatient admissions with the diagnosis of DM, and with a prescription for insulin, analogues, or antidiabetes medications for more than 30 days; 3 or more outpatient visits with a diagnosis of DM and with a prescription for insulin, analogues, or antidiabetes medications for more than 30 days.

### 2.3 Statistical analysis

The demographic and clinical data for the HAART and non-HAART cohorts were analyzed by 2-sample *t* test for continuous variables and by chi-square test for categorical variables. Kaplan–Meier methods were used to determine the cumulative incidence of DM in both cohorts and the differences between the 2 cohorts were tested using the log-rank test. Death prior to the development of DM was considered a competing risk event. The HAART cohort and the non-HAART cohort were compared to evaluate the varying risks of developing DM with the use of stratified Fine and Gray regression hazards model, adjusting for age, gender, and comorbidities.^[16]^ To examine whether the main findings met different assumptions, we performed sensitivity analyses, with the use of Fine and Gray regression model on subgroups classified by age, gender, and comorbidities. All data management and analyses were performed using the SAS System (version 9.4; SAS Institute, Cary, NC).

## 3 Results

### 3.1 Baseline characteristics of HAART and non-HAART cohorts

Demographic and clinical characteristics and observation duration of the 2 study cohorts are presented in Table 1. The mean age of the 2 cohorts was 32.9 years (standard deviation [SD], 8.6), with the majority being male (91.9%). The mean duration of HAART exposure during the observation period in the HAART cohort was 332 days per year. Individuals in the HAART cohort had a higher prevalence of hypertension, gout, chronic HBV infection, and cancer. On the contrary, the prevalence of HCV infection was higher in the non-HAART cohort than in the HAART cohort. Similarly, the mortality rate was also higher in the non-HAART cohort. After a mean observation of 7.5 years (SD, 3.3) and 8.1 years (SD, 3.3), a total of 44 (0.9%) and 99 (2.1%) cases of DM were diagnosed in the non-HAART cohort and HAART cohort, respectively (*P* < .001).

**Table 1:**
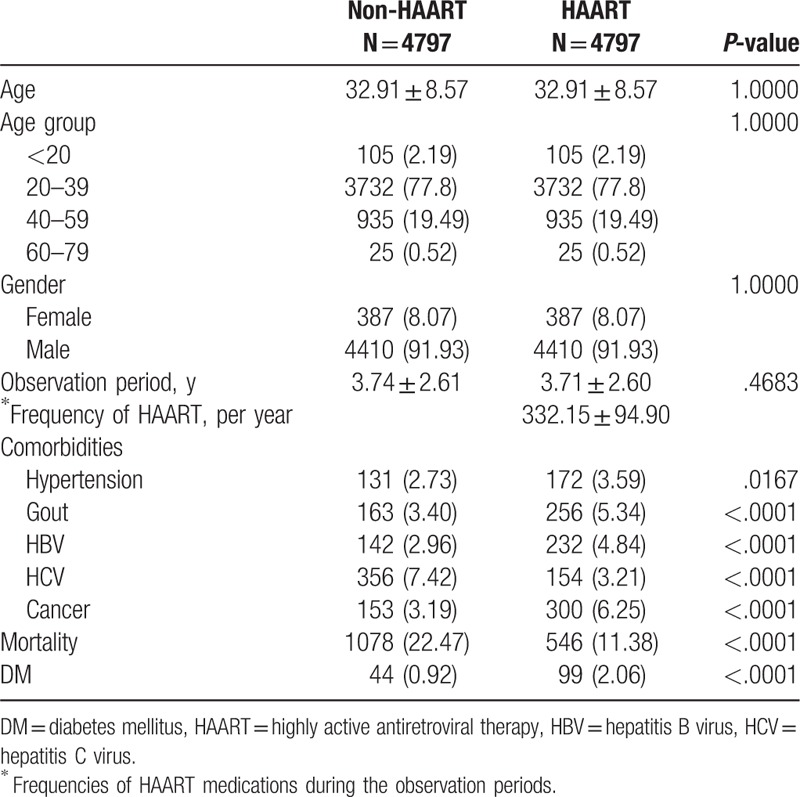
Baseline characteristics of HAART and non-HAART cohorts.

### 3.2 Ten-year cumulative incidence of DM among HIV-infected patients

**Figure 2:**
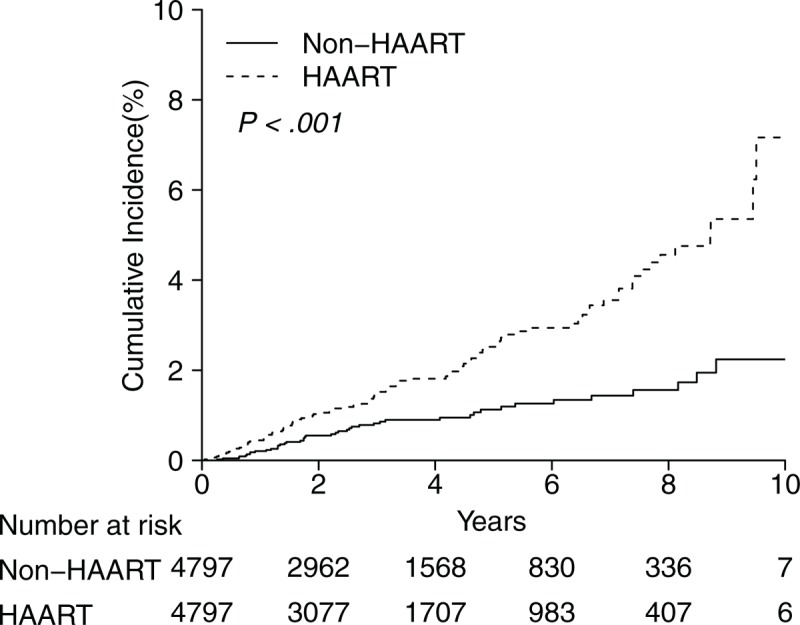
Ten-year cumulative incidence of developing diabetes mellitus.

The cumulative incidences of DM in the HAART and non-HAART cohorts are shown in Figure 2. Ten-year cumulative incidence of DM among patients in the HAART cohort was 7.16% (95% confidence interval [CI], 4.30%–10.03%), which was significantly higher than that among those in the non-HAART group (2.24%; 95% CI, 1.28%–3.20%) (*P* < .001), with a difference in the 10-year cumulative incidence of 4.92%. The average annual incidence was 0.72% in HAART cohort and 0.22% in the non-HAART.

### 3.3 Multivariable analysis

The results of subdistribution hazard ratio (sHR) of DM that were estimated after adjustments made for age, gender, and comorbidities are showed in Table 2. Compared with non-HAART cohort, HAART cohort had a higher risk of DM, with an sHR of 2.39 (95% CI, 1.65–3.45). Comorbidities of hypertension (sHR = 5.27; 95% CI, 3.21–8.65), gout (sHR = 2.39; 95% CI, 1.38–4.16), and HCV infection (sHR = 2.43; 95% CI, 1.28–4.61) were statistically significantly associated with a higher risk of DM.

**Table 2:**
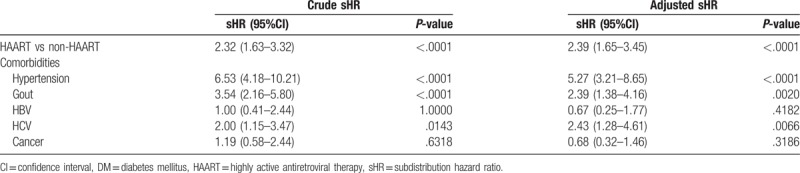
Prediction for DM: adjusted age, gender, comorbidities.

### 3.4 Multivariable stratified analysis

**Figure 3:**
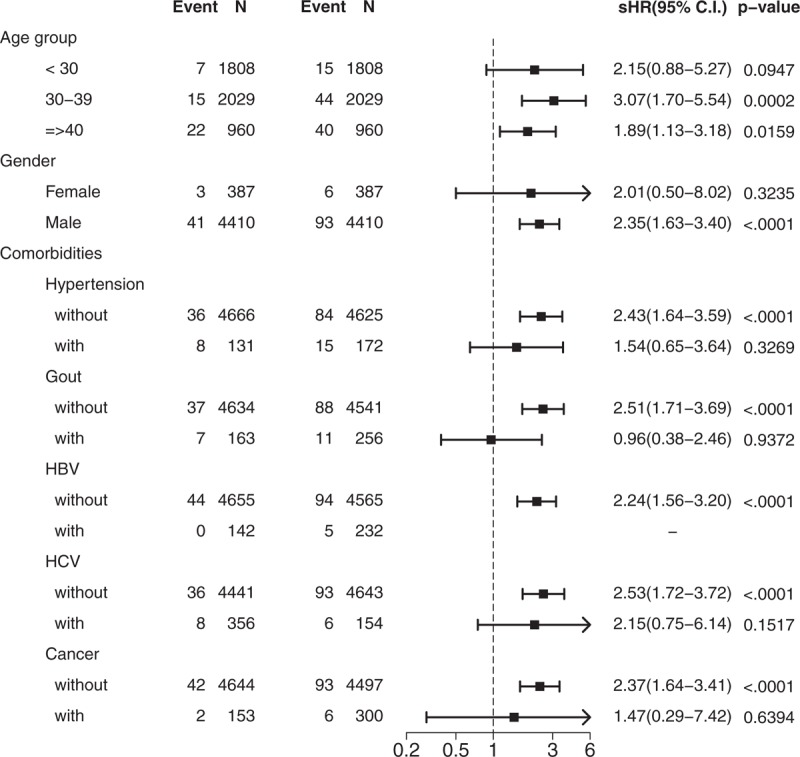
Forest plot for sub-groups.

The results of adjusted HR of DM for the HAART cohort stratified by the subgroups of age, gender, comorbidities of hypertension, gout, HBV, HCV, and cancer are shown in Figure 3, which indicates that HAART remained significantly associated with risk of DM, but only in those without hypertension (sHR = 2.43; 95% CI, 1.64–3.59), gout (sHR = 2.51; 95% CI, 1.71–3.69), and HCV infection (sHR = 2.53; 95% CI, 1.72–3.72).

## 4 Discussion

In this nationwide population-based study by including all HIV-infected patients in Taiwan, we found that exposure to HAART was associated with an increased risk of DM among HIV-infected patients, particularly in those patients without pre-existing hypertension, gout, or HCV infection. This is to our knowledge the largest cohort study of DM, in which the study population comprised non-Caucasian HIV-infected patients.

In our study, the annual incidence (0.22%) of DM in the non-HAART cohort was similar to that of the general population in Taiwan.^[17]^ According to a recently published national cohort study that was conducted in Taiwan in the period of 1999 to 2004, the annual incidence of DM among individuals aged 20 to 39 years was 0.15% to 0.19%.^[17]^ Our HIV-infected individuals were observed to have a comparable DM incidence to the general population in similar age groups. Our findings are also similar to the results of several previous cohort studies. In a Danish HIV cohort study conducted in the period of 1996 to 1998, the annual incidence of DM was 0.1% in the general population and 0.27% in the non-HAART cohort.^[18]^ However, several cohort studies revealed higher rates of DM in both HIV-uninfected and HIV-infected population than that of our HIV-infected cohort.^[11,12,19]^ In a South Carolina Medicaid cohort, the annual incidence of DM was 1.36% in the HIV-uninfected population and 1.12% in the non-HAART HIV-infected cohort.^[19]^ In the multicenter AIDS cohort study (MACS), the annual incidence of DM was 1.4% in the HIV-uninfected population and 1.7% in the non-HAART HIV-infected cohort.^[11]^ In the women's interagency HIV study (WIHS), the annual incidence of DM was 1.96% in the HIV-uninfected population and 1.53% in the non-HAART HIV-infected cohort.^[12]^ The lower incidence in our study is probably due to the fact that our study included patients of significantly younger age (32 years in our study vs 39 years in the South Carolina Medicaid cohort, 46–50 years in MACS, and 39 years in WIHS). In addition, the definition of DM used in our study included physician's diagnosis plus use of antidiabetic medications, which might have underestimated the incidence of DM, while the definitions of DM used in the 3 previous studies was diagnosis code of DM, the prescription of an antidiabetic medication, or physician's diagnosis. Moreover, our study excluded those with DM prior to or within the 365th day following the initial date of HIV diagnosis because of difficulty in establishing the relationship between HIV infection and the subsequent DM.

Our study found that HAART, which mainly consisted of 2 NRTIs plus nNRTI or PIs during the study period, was an independent risk factor for DM. PIs can induce insulin resistance through the inhibition of glucose transporter type 4. Moreover, NRTIs have been known to cause mitochondrial dysfunction in adipocytes and contribute to lipoatrophy and insulin resistance.^[8,20,21]^ These phenomena may explain the association of long-term exposure to HAART that predominantly consisted of 2 NRTIs plus nNRTI or PIs with the development of DM. However, the incidence of DM in the literature may vary due to differences in study population, inclusion/exclusion criteria, and HAART and its exposure duration. In our study, the annual incidence of diabetes was 0.72% in the HAART cohort. Compared to previous cohort studies, the incidence of DM in our HAART cohort is lower than those reported in the APROCO-COPILOTE cohort (1.41% after HAART initiation),^[14]^ a recent case–control study in Taiwan (1.31% in HIV-infected patients receiving HAART),^[22]^ WIHS (2.50% in PI-containing HAART, and 2.89% in non-PI containing HAART),^[12]^ and MACS (4.7% in the HAART group).^[11]^ Other than the younger patients population in our study (32 years in our study vs 51 years in the Taiwan case–control study, 39 years in WIHS, and 46 years in MACS), the diagnostic definition of DM used and types and durations of HAART the patients included in those studies might have been exposed to might also be contributory to the differences in the rates of DM observed.

We found that HCV infection was associated with an increased risk of DM in HIV-infected patients. HCV infection can induce insulin resistance and hence leads to development of DM.^[23]^ Two meta-analysis studies have shown that HCV infection increased the risk of DM by 1.4- to 1.6-fold compared to HCV-seronegative controls.^[24,25]^ Antiviral therapy for HCV infection might decrease the risks of renal and cardiovascular complications among the diabetic patients.^[26]^ Among HIV-infected patients, HCV infection was also shown to increase the risk of DM by 1.5-fold in the Veterans Aging Cohort Study,^[27]^ and by 1.8-fold in a meta-analysis study.^[24]^ In our study, the annual incidence of DM in HAART cohort was 0.43% in HCV-infected patients, 0.22% in HCV-uninfected patients, 0.20% in HCV-infected patients not receiving HAART, and 0.09% in HCV-uninfected patients not receiving HAART. Overall, the rate of DM in HIV/HCV-coinfected patients was 2.43-fold higher than that in HIV-monoinfected patients.

Hyperuricemia has been shown to act as a sympathetic activator, and is associated with renin–angiotension–aldosterone system activation, oxidative stress, and crystal-induced inflammatory response.^[28]^ Data from previous studies revealed that hyperuricemia mediates increased insulin resistance and decreased insulin release, and eventually leading to DM.^[29–31]^A cohort study using the US health care utilization database, in which gout was associated with a 1.45-fold increased risk of DM compared to patients with osteoarthritis.^[32]^ Moreover, the age-adjusted standardized incidence ratio of DM in patients with gout was 2.59.^[33]^ The association between gout and DM in HIV-infected patients has not been previously investigated. In this study, we found that gout was associated with a 2.39-fold increased risk of DM in HIV-infected patients. Whether the association implies causal relationship between derangement of uric acid metabolism and development of DM warrants further investigation.

Our study revealed that HAART was associated with an increased risk of DM, but only in patients without comorbidities of hypertension, gout, or HCV infection. In HIV-infected patients, in addition to long-term exposure to HAART, several comorbidities have been shown to be associated with DM, including hypertension and HCV.^[8]^ Independent of HAART, the association between comorbidities of hypertension, gout, and HCV infection with DM was strong in our study. Although only a few cases of DM remained in our subgroups analyses, we found that HAART did not further increase the risk of DM in HIV-infected patients with these comorbidities. Our study was inconsistent with the study from Veterans Affairs (VA) administrative database,^[34]^ which revealed that HCV infection was associated with a 1.39-fold increased the risk of DM in the HAART era, but not in the pre-HAART era. The finding might be explained by the shorter survival period in the patients included in the pre-HAART era. In the modern era of treat-all and rapid initiation of HAART, more long-term studies are needed to investigate the incidence of DM among HIV-infected patients without any comorbidities who are likely to enjoy similar longevity to HIV-uninfected individuals.

There are limitations in our studies and cautions are needed in interpretation of our findings. First of all, the database used in this study did not contain personal information on lifestyle behaviors, such as smoking, alcohol drinking, dietary habits, and leisure-time physical activity, family history, body-mass index, and laboratory parameters including CD4 cell count, and HIV virus load, which may act as confounders. Even though the HAART cohort had a more advanced HIV status compared to the non-HAART cohort, suggesting the risk of DM should have been higher in all of the HAART subgroups, the multivariable stratified analysis revealed that the impact on the DM risk was less severe in patients with comorbidities of hypertension, gout, and HCV (albeit with small patient numbers in the subgroups). This finding further supports an association between HAART and risk of DM for those patients without hypertension, gout, or HCV infection. Another limitation is the incidence of DM in our study may be underestimated due to the rigorous definition of diabetes (physician's diagnosis plus antidiabetic medication use). The patients with DM who did not receive antidiabetes medications would not be included in this study.

In conclusion, although the annual incidence of DM HIV-infected patients not receiving HAART was similar to that of the general Taiwanese population, exposure to HAART increased the risk of DM in the HIV-infected patients receiving HAART who had no pre-existing hypertension, gout, or HCV infection.

## Acknowledgments

The authors thank grants from the Ministry of Science and Technology, The Executive Yuan of Taiwan (MOST 104-3011-E-006-003-), National Science Council (NSC 101-3114-Y-006-001), and National Cheng Kung University Hospital (NCKUH 101-07002) for the support.

## Author contributions

**Conceptualization:** Shih Ping Lin, Chun-Ying Wu.

**Data curation:** Shih Ping Lin, Chun-Ying Wu, Chang-Bi Wang.

**Formal analysis:** Shih Ping Lin, Chun-Ying Wu, Chang-Bi Wang.

**Investigation:** Shih Ping Lin, Chun-Ying Wu, Chang-Bi Wang, Tsai-Chung Li.

**Methodology:** Shih Ping Lin, Chun-Ying Wu.

**Project administration:** Shih Ping Lin, Chun-Ying Wu.

**Supervision:** Chun-Ying Wu, Tsai-Chung Li, Nai-Ying Ko, Zhi-Yuan Shi.

**Validation:** Shih Ping Lin, Tsai-Chung Li.

**Visualization:** Shih Ping Lin, Tsai-Chung Li.

**Writing – original draft:** Shih Ping Lin.

**Writing – review & editing:** Shih Ping Lin.
